# Supplementary material for: Identification and characterization of diverse OTU deubiquitinases in bacteria
Source: EMBO J. 2020 Jun 22;39(15):e105127. doi: 10.15252/embj.2020105127 (PMC7396840; doi:10.15252/embj.2020105127)
Supplement: Supplementary file 1 — Expanded View Figures PDF [file EMBJ-39-e105127-s001.pdf]

Expanded View Figures

Figure EV1. Prediction and validation of OTU DUBs from bacteria.

A Workflow illustrating the process used for bioinformatic prediction of bacterial OTU domains, followed by their manual curation. Representative output from the Phyre2 curation is shown for the predicted EschOTU active site.

B Table presenting prediction scores for type III and type IV secretion signals using the pEFFECT (Goldberg *et al*, 2016) and S4TE 2.0 (Noroy *et al*, 2019) prediction approaches, respectively. For pEFFECT predictions, prediction reliability scores above 50 or above 80 are associated with 87% or 96% accuracy, respectively. For S4TE 2.0, a prediction score above 72 is associated with a 98% sensitivity. Organisms that lack one of the secretion systems are marked as not applicable (NA).

C Full fluorescent Ub substrate cleavage data for all bacterial OTUs following Ala substitution at each member of the predicted catalytic triad. These data were collected in parallel with those presented in Fig 1G, and the WT dataset is shown again for clarity. The rise above 100% observed with BurkOTU is indicative of a noncovalent interaction.

© 2020 The Authors

A

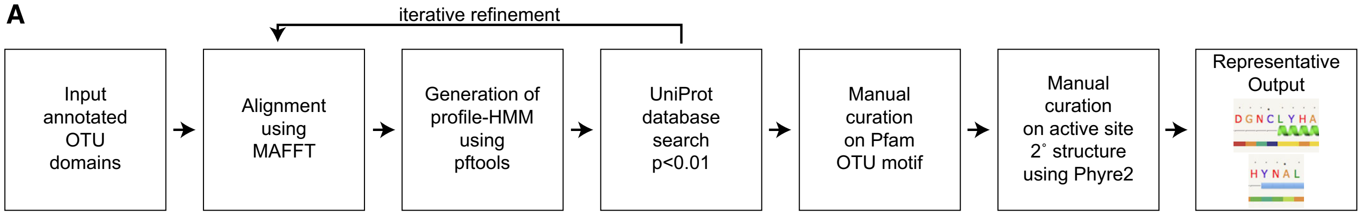

B

	EschOTU	ceg7	BurkOTU	ChlaOTU	RickOTU	wPipOTU	wMelOTU	ceg23
Type III pEFFECT	Yes (91)	NA	Yes (61)	Yes (89)	NA	NA	NA	NA
Type IV S4TE 2.0	NA	Yes (119)	No (53)	NA	Yes (114)	Yes (85)	Yes (81)	Yes (129)

C

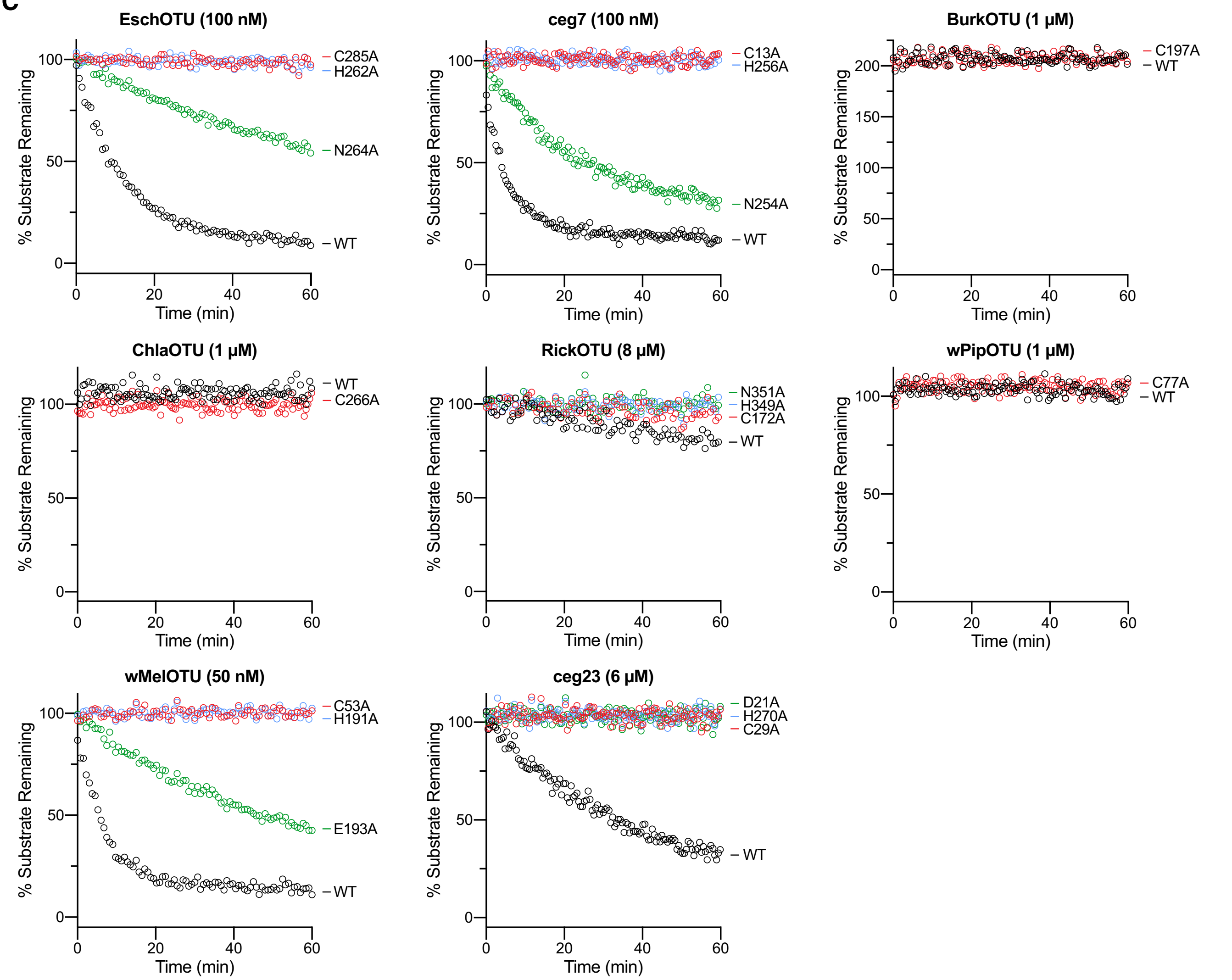

Figure EV1.

 © 2020 The Authors

Figure EV2. Substrate specificity profiling of bacterial OTU DUBs.

A Corrected Ub/Ub-like substrate specificity assays for all bacterial OTUs.

B Ub chain specificity assays for EschOTU, ceg7, ChlaOTU, RickOTU, wPipOTU, and ceg23 toward the eight diUb linkages. Reaction samples were quenched at the indicated timepoints, resolved by SDS–PAGE, and visualized by Coomassie staining.

C K11 diUb cleavage assay for BurkOTU WT and Ala-substituted catalytic triad mutants. Reaction samples were quenched at the indicated timepoints, resolved by SDS–PAGE, and visualized by Coomassie staining.

© 2020 The Authors

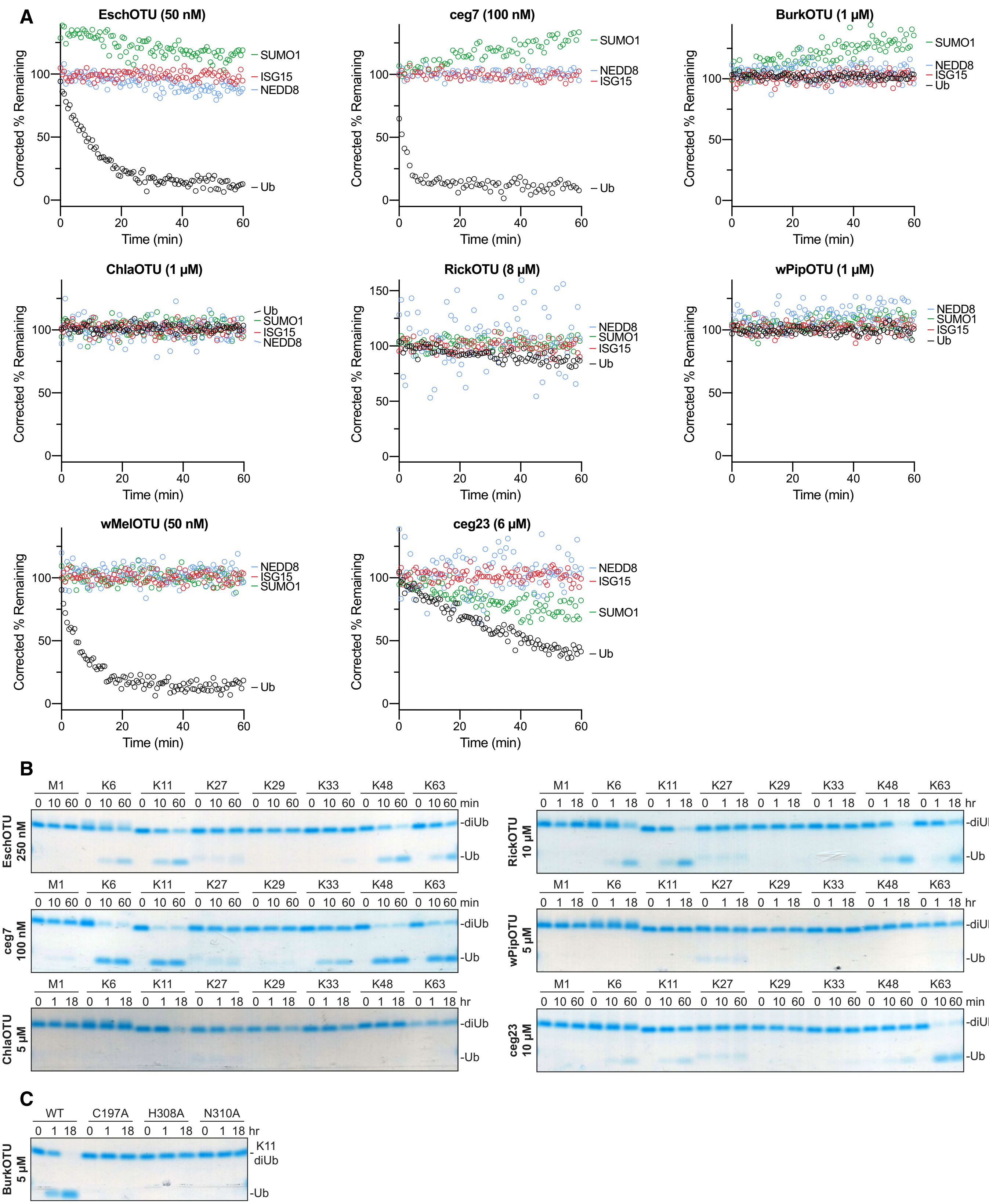

Figure EV2.

 © 2020 The Authors

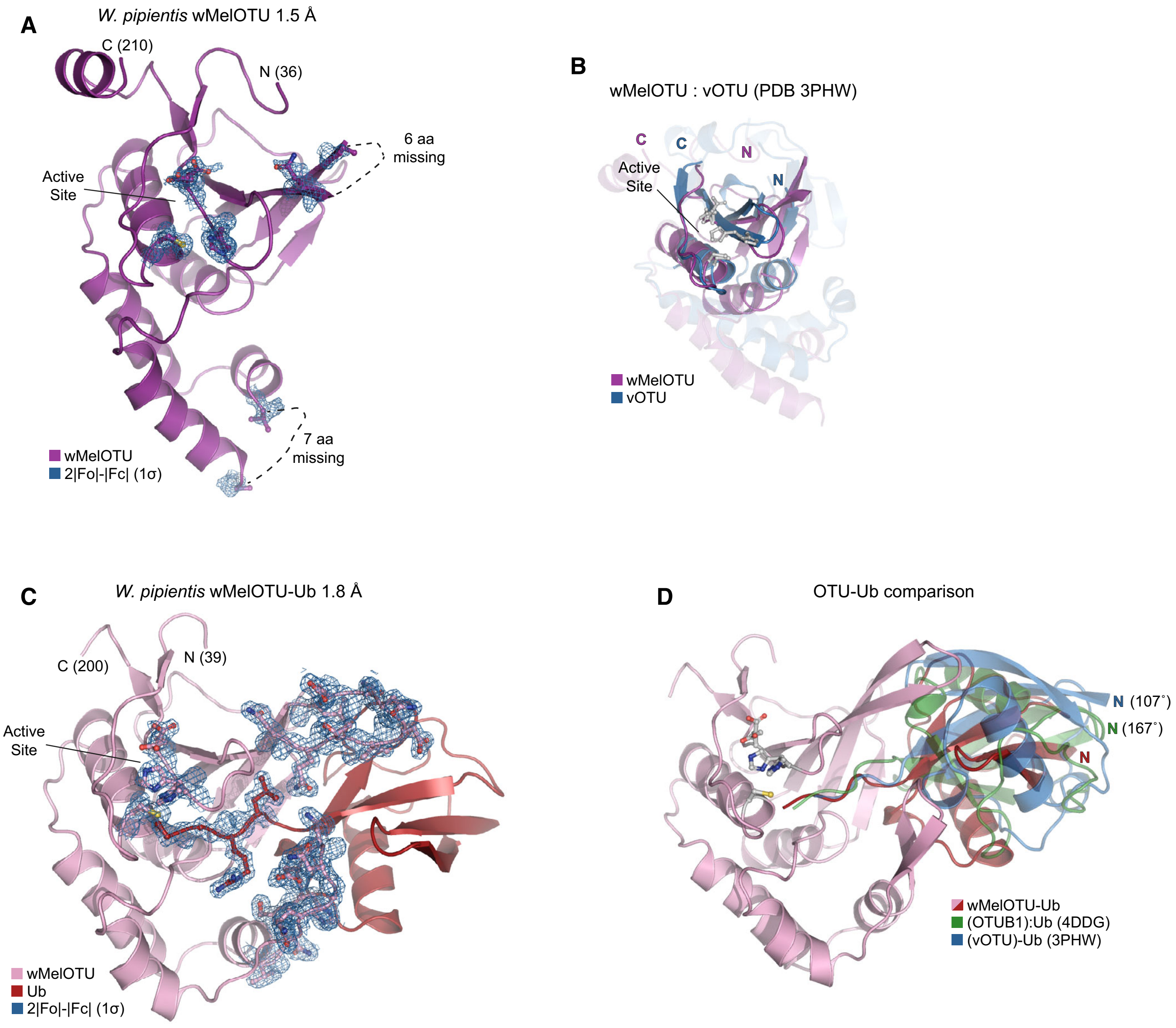

Figure EV3. wMelOTU structure reveals novel Ub embrace mechanism.

A Cartoon representation of the 1.5 Å *Wolbachia pipientis* wMelOTU crystal structure with representative 2|F_o|−|F_c| electron density contoured at 1σ. Electron density is shown for catalytic triad residues as well as either edge of regions lacking interpretable density.

B Structural alignment of the core OTU folds (central β-sheet and two supporting α-helices) from CCHFV vOTU (blue, PDB 3PHW) and wMelOTU (purple). Surrounding regions are less well conserved and shown as semi-transparent.

C Cartoon representation of the 1.8 Å *W. pipientis* wMelOTU-Ub crystal structure with representative 2|F_o|−|F_c| electron density contoured at 1σ. Electron density is shown for the wMelOTU catalytic triad residues, the Ub C-terminus, the two wMelOTU β-hairpin regions that form the Ub embrace.

D Cartoon representation of the wMelOTU-Ub crystal structure (pink/red) overlaid with the bound Ub molecules from the OTUB1:Ub structure (green, PDB 4DDG) and the CCHFV vOTU-Ub structure (blue, PDB 3PHW). The OTUB1- and vOTU-bound Ub molecules are rotated by 167° and 107°, respectively, in relation to the wMelOTU-bound Ub.

© 2020 The Authors

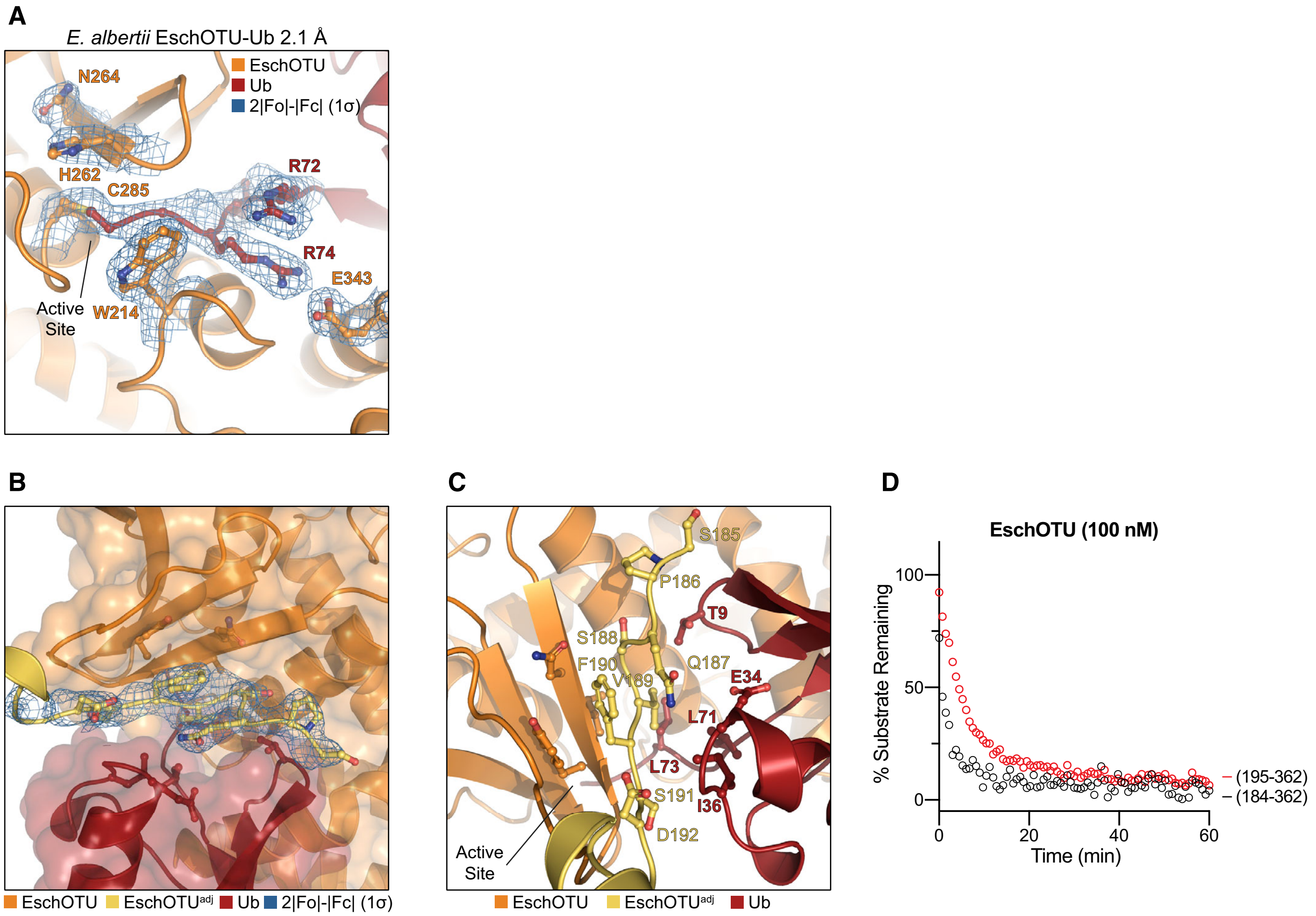

Figure EV4. EschOTU structure shows altered sequence topology.

A Detailed view of the active site in the EschOTU-Ub crystal structure with representative $2|F_o|-|F_c|$ electron density contoured at 1σ. Electron density is shown for the EschOTU active site as well as the Ub C-terminus and EschOTU residues that coordinate it.

B Transparent surface representation of the EschOTU-Ub crystal structure (orange/red) showing insertion of the N-terminus from a symmetry-related molecule (yellow) with $2|F_o|-|F_c|$ electron density contoured at 1σ.

C Detailed view of the EschOTU N-terminal insertion from a symmetry-related molecule (yellow), and contacts to EschOTU (orange) and Ub (red).

D Ub-KG(TAMRA) cleavage assay showing little effect on activity following removal of the N-terminal region (residues 184–194).

 © 2020 The Authors

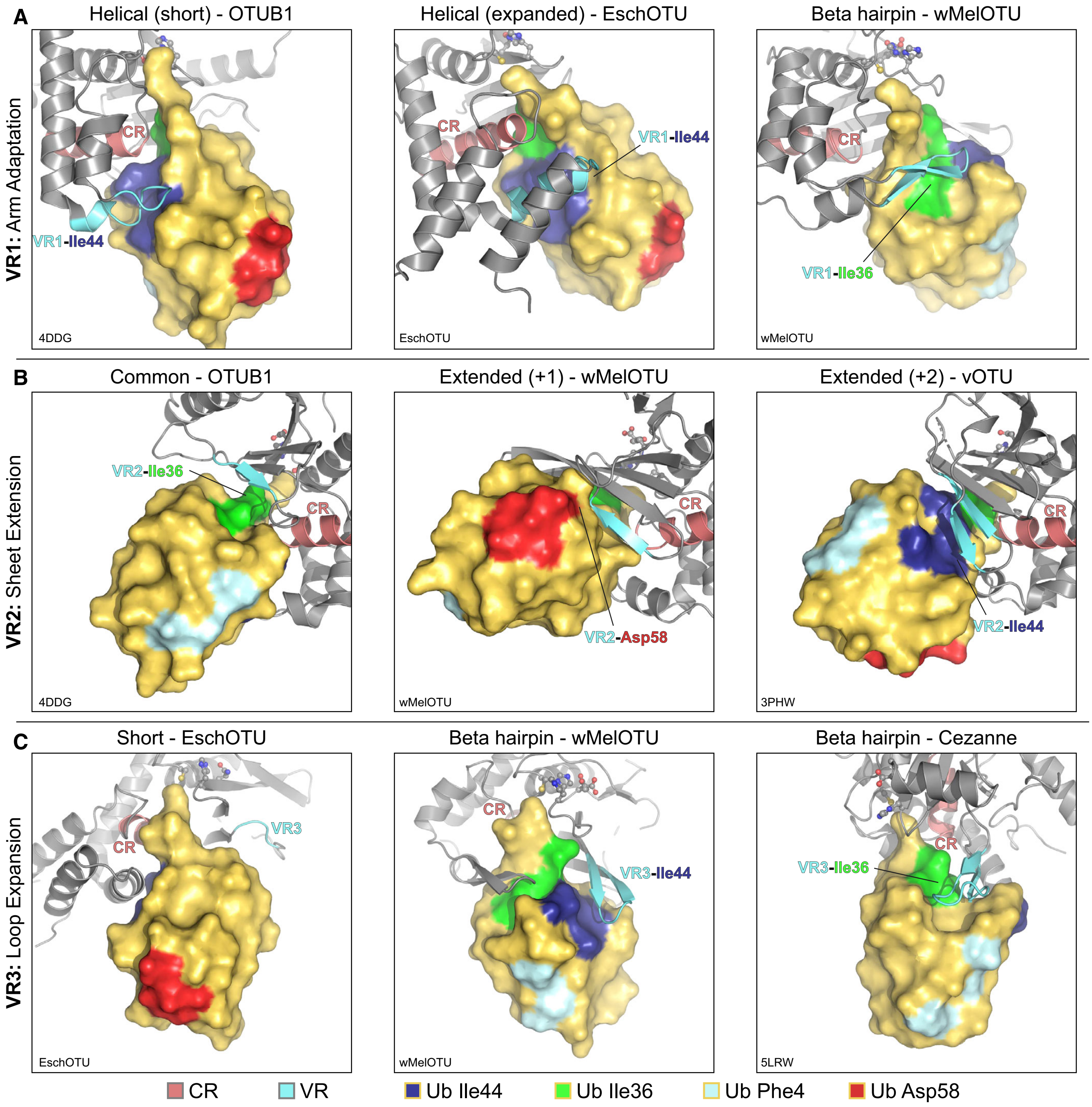

Figure EV5. A framework for understanding the S1 site of OTU domains.

A Examples of Ub interaction surfaces contacted by OTU VR1 arm regions in the S1 site. OTUB1 (left) and EschOTU (center) contact the Ub Ile44 hydrophobic patch (blue), while wMelOTU (right) contacts the Ile36 hydrophobic patch (green).

B Examples of Ub interaction surfaces contacted by OTU VR2 β-sheet edges in the S1 site. OTUB1 (left) contacts the Ub Ile36 hydrophobic patch (green), while wMelOTU (center) reaches to the Asp58 acidic patch (red) and CCHFV vOTU (right) contacts the Ile44 hydrophobic patch (blue).

C Examples of Ub interaction surfaces contacted by OTU VR3 loops in the S1 site. EschOTU (left), with its short VR3, makes no Ub contacts, while wMelOTU (center) contacts the Ub Ile44 hydrophobic patch (blue) and Cezanne (right) contacts the Ile36 hydrophobic patch (green).

© 2020 The Authors

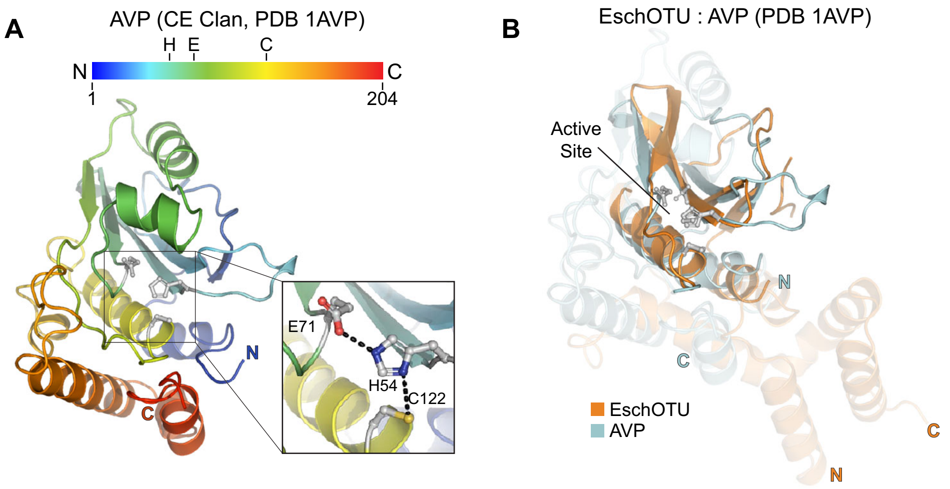

Figure EV6. Cross-kingdom structural analysis of the OTU fold.

A Cartoon representation of the CE clan, human adenovirus 2 proteinase crystal structure (PDB 1AVP) colored in a rainbow gradient from N- to C-terminus. The catalytic triad residues are marked on both the structure and the linear color gradient above, showing their positions with respect to each other and the overall sequence.

B Structural alignment of the core protease folds (central β-sheet and two supporting α-helices) from human adenovirus proteinase 2 of the CE clan (light blue, PDB 1AVP) and EschOTU (orange). Surrounding regions are less well conserved and shown as semi-transparent.

 © 2020 The Authors
